# Supplementary material for: Patterns of Intron Gain and Loss in Fungi
Source: PLoS Biol. 2004 Nov 30;2(12):e422. doi: 10.1371/journal.pbio.0020422 (PMC532390; doi:10.1371/journal.pbio.0020422)
Supplement: Table S1 — Also available at http://genes.mit.edu/NielsenEtAl/. (4.3 MB ZIP). [file pbio.0020422.st001.zip › NielsenEtAl/html/592.html]

AN6525.1.NCU03813.1.MG04034.1.FG06127.1


```
 CLUSTAL W (1.82) Multiple Sequence Alignments - Introns Inserted


Sequence 1: NCU03813.1	375 aa
Sequence 2: MG04034.1	363 aa
Sequence 3: FG06127.1	365 aa
Sequence 4: AN6525.1	377 aa
Alignment Length: 392 aa
Number Identitical Residues: 276 aa
Alignment Score (without introns) 11643


MG04034.1 	-~----MVLYD~GGQHAKD0VPELLGTTENELGIRKWLEDQGHTLVTTSDKDGENSTFDK
NCU03813.1	M0VKVLAVLYD~GGKHGEE0VPELLGTIQNELGLRKWLEDQGHTLVTTCDKDGENSTFDK
FG06127.1 	M0VKVLAVLYD~GGQHAKD0QPLLLGTTENELGIRKWLEDQGHTLVTTSDKDREGSKFDE
AN6525.1  	-~----MVLYD0GGSHAKD~QPGLLGTTENELGIRKWIEEQGHTLVTTSDKDGENSTFDK
          	       **** **.*.::  * **** :****:***:*:********.*** *.*.**:

MG04034.1 	ELEDAEIIITTP2FHPGYLSAERLARAKKLKLTVTAGIGSDHVDLNAANKTNGGITVAEV
NCU03813.1	ELEDAEIIITTP~FHPGYLTAERLARAKKLKLAVTAGIGSDHVDLNAANKTNGGITVAEV
FG06127.1 	ELEDAEIIITTP~FHPGYLTAERLAKAKKLKLAVTAGIGSDHVDLNAANKTNGGITVAEV
AN6525.1  	ELVDAEVIITTP2FHPGYLTAERLAKAKNLKLAVTAGIGSDHVDLDAANKTNGGITVAEV
          	** ***:***** ******:*****:**:***:************:**************

MG04034.1 	TGSNVVSVAEHVLMTILVLVRNFVPAHEMIQAGEWDVAGAAKNEYDLEGKVVGTVAVGRI
NCU03813.1	TGSNVVSVAEHVLMTILVLVRNFVPAHEQIQEGRWDVAEAAKNEFDLEGKVVGTVGVGRI
FG06127.1 	TGSNVVSVAEHVLMTILVLIRNFVPAHEQIEAGEWDVAHAAKQEFDLEGKVVGTVAVGRI
AN6525.1  	TGSNVVSVAEHVVMTILLLVRNFVPAHDQIRNGDWNVAAVAKNEFDLENKVVGTVGVGRI
          	************:****:*:*******: *. * *:** .**:*:***.******.****

MG04034.1 	GERVLRRLKPFDCKELLYYDYQPLAPEVEKEIGCRRVDNLEEMLAQCDVVTINCPLHEKT
NCU03813.1	GERVLRRLKPFDCKELLYYDYQPLSAEKEAEIGCRRVADLEEMLAQCDVVTINCPLHEKT
FG06127.1 	GERVLRRLKPFDCKELLYFDYQPLSPEAEKEIGCRRVDTLEEMLAQCDIVTINCPLHEKT
AN6525.1  	GERVLRRLKPFDCKELLYYDYQPLRPEVEKEIGARRVDSLEEMVSQCDVVTINCPLHEKT
          	******************:***** .* * ***.***  ****::***:***********

MG04034.1 	RGLFNKDLISKMK-----------------KG1SWLVNTARGAIVVKEDVAEALKTGHLR
NCU03813.1	QGLFNKELISKMK-----------------KG~SWLVNTARGAIVVKEDVAEALKSGHLR
FG06127.1 	KGMFNKDLISKMK-----------------KG~SYLVNTARGAIVVKEDVAAALKSGHLA
AN6525.1  	RGLFNKELISKMKPGKSALLYLIIPMLMYHKG~SWLVNTARGAIVVKEDVAEALKSGHLR
          	:*:***:******...::      .     ** *:**************** ***:*** 

MG04034.1 	GYGGDVWFPQPAPKDHPLRYAKNPFGGGNAMVPHMSGTSLDAQKRYADGTKAILESYLSG
NCU03813.1	GYGGDVWFPQPAPQDHPLRYAKNPFGGGNAMVPHMSGTSLDAQKRYAAGTKAIIESYLSG
FG06127.1 	GYGGDVWDHQPAPKEHPLRNAKNNWGGGNAMVPHMSGTSLDAQIRYANGTKAIIDSYLSG
AN6525.1  	GYGGDVWFPQPAPKEHPLRYAEHPWGGGNATVPHMSGTSIDAQIRYANGTKAILDSYFSG
          	*******  ****::**** *:: :***** ********:*** *** *****::**:**

MG04034.1 	KLDYRPQDLIVHAGDYATKAYGERAKITKA-------
NCU03813.1	KHDYRPEDLIVYGGDYATKSYGERERAKAAAAAAKSA
FG06127.1 	RHDYNPHDLIVHQGDYATKAYGQREKK----------
AN6525.1  	RFDYQPQDLIVHGGDYATKAYGQREKK----------
          	: **.*.****: ******:**:* :
```
